# Modulatory effects of polyherbal mixture on the immuno-antioxidant capacity and intestinal health of chicks infected with *Escherichia coli* O78

**DOI:** 10.1016/j.psj.2025.105156

**Published:** 2025-04-12

**Authors:** Qian Qu, Mengjie Liu, Yifan Hu, Gengxiong Huang, Zhaoying Xuan, Jianchi Lun, Xiaoli Chen, Weijie Lv, Shining Guo

**Affiliations:** a College of Veterinary Medicine, South China Agricultural University, Guangzhou, China; b School of Animal Science and Technology, Foshan University, Foshan, China; c Guangdong Technology Research Center for Traditional Chinese Veterinary Medicine and Natural Medicine, Guangzhou, China

**Keywords:** Polyherbal mixture, Chick, *Escherichia coli* O78, Immune response, intestinal health

## Abstract

A total of 180 one-day-old white-feathered broiler chicks were selected and randomly divided into 4 treatments, namely the control group (**CON**), *Escherichia coli* groups (***E. coli***), 2 g/kg polyherbal mixture group (**PHM2**), and the 4 g/kg polyherbal mixture group (**PHM4**). The CON and *E. coli* groups were fed a basal diet, while the PHM2 and PHM4 groups were fed the basal diet supplemented with 2 g/kg and 4 g/kg PHM, respectively. Each group had 3 replicates, with 15 broilers per replicate. On day 17 of the experiment, broilers in the *E. coli*, PHM2, and PHM4 groups were intraperitoneally injected with 0.8 mL of 1 × 10^8^ CFU/mL of *E. coli* O78. Broilers in the control group received an equivalent volume of saline. Chicks were euthanized 48 h postinjection for collecting serum, liver, spleen, jejunum, ileum, ileal mucosa, and cecal contents. Our results showed that PHM significantly reversed the weight loss and decreased the diarrhea rate and the mortality of chicks caused by *E. coli* infection (*P* < 0.05). In the serum of chicks infected with *E. coli*, PHM significantly enhanced the antioxidant capacity (*P* < 0.05), increased the levels of immunoglobulins and anti-inflammatory cytokines (*P* < 0.05), and decreased the concentrations of proinflammatory cytokines (*P* < 0.05). Meanwhile, PHM also promoted the mRNA expression of antioxidant-related genes and decreased the expression of proinflammatory cytokines and apoptosis-related genes in the liver, spleen, jejunum, and ileum (*P* < 0.05). In addition, PHM repaired the intestinal barrier and injury to further reduce the serum concentrations of d-lactate (**DAO**) and lipopolysaccharide **(LPS**) (*P* < 0.05). More importantly, PHM significantly regulated the composition of cecal microbiota, especially by up-regulating the relative abundance of beneficial bacteria, including *Faecalibacterium, Bacteroides, Butyricicoccus,* and *Lactobacillus*, and down-regulating the relative abundance of pathogenic bacteria, including *Enterococcus, Escherichia,* and *Shigella* (*P* < 0.05). These beneficial bacteria were significantly positively correlated with antioxidant capacity and intestinal barrier function, while pathogenic bacteria were significantly positively correlated with proinflammatory cytokines (*P* < 0.05). In conclusion, PHM may be a potential preventive strategy for *E. coli-*infected poultry, which is closely related to its modulation of gut microbiota.

## Introduction

Immune stress leads to damage to the intestinal barrier and digestive disorders in poultry, triggering various inflammatory diseases, reducing their production performance, and even leading to death, resulting in huge economic losses (Zhang et al., 2020). Avian pathogenic *Escherichia coli* (**APEC**) is the main Gram-negative bacteria responsible for immunosuppressive diseases or environmental stress and is one of the most common pathogens in poultry production, causing sepsis, hemorrhagic sepsis, and enteritis (Kathayat et al., 2021; Ren et al., 2024). Gram-negative bacteria release a large amount of endotoxin (such as LPS) during the propagation and growth of infected hosts, which is an important component of pathogenic microorganisms to damage the body (Park and Lee, 2013). Although *E. coli* is an extraintestinal pathogen, its colonization, and growth mainly occurs in the intestinal lumen, causing severe diarrhea, reduced growth performance, and feed intake, especially in chicks with insufficient immune function growth and development (La Ragione and Woodward, 2002; Mellata, 2013; Hashem et al., 2022).

The diseases associated with APEC are primarily caused by environmental factors and host susceptibility, resulting in significant economic losses to the poultry industry (Xu et al., 2024). In recent years, herbal products have been used in poultry diets due to their growth-promoting and natural immuno-stimulating effects (Kuralkar and Kuralkar, 2021). Liu et al*.* showed that adding Chinese herbal medicine to the feed of laying hens improved their production performance, egg quality, antioxidant capacity, immunity, and intestinal health (Liu et al., 2023). Chinese herbal is a strong stimulant of poultry digestion and immunity because of its rich active ingredients, including organic acids, polyphenols, flavonoids, polysaccharides, etc., and has a positive effect on poultry (Liu et al., 2022; Abo Ghanima et al., 2023; Rafeeq et al., 2023). Many studies have shown that Chinese herbal medicine reduced oxidative stress through Nrf2 signaling and inflammation through NF-κB, repaired intestinal barriers through tight junction proteins, and enhanced immunity by reducing apoptosis, which is disrupted in poultry infected with *E. coli* (Wang et al., 2016; Wan et al., 2021, 2023).

The symbiotic relationship between the microbiota and the host is mutually beneficial, with the gut microbiota assisting the host to absorb nutrients and the host providing nutrients and suitable habitat for the gut microbes (Guo et al., 2022). Gut microbiota forms a multi-layer microbial barrier in the gut, which plays a crucial role in intestinal defense and immune function regulation (Murga-Garrido et al., 2021). Once the body is stimulated, gut microbiota will suffer drastic changes. When poultry is attacked by bacteria, viruses, etc., the integrity of the intestine will be damaged, and intestinal development, digestion, and absorption of nutrients will be affected, accompanied by changes in intestinal microbial composition, which also occurs in *E. coli-*infected poultry (Gonzalez-Quilen et al., 2020; Li et al., 2024, 2024). More importantly, many traditional Chinese medicines (**TCM**) have been reported to improve bacterial, viral, and other diseases by regulating gut microbiota (Wu et al., 2018; Li et al., 2022).

In our previous research, a polyherbal mixture (**PHM**) of five TCMs, including *Portulaca oleracea L., Radix Sophorae Flavescentis, Thalictrum glandulosissimum, Terra flava usta*, and *Pogostemon cablin*, significantly improved growth performance, regulated immune function, reduced oxidative stress, and repaired intestinal damage (Liu et al., 2023). Therefore, we wanted to further explore whether PHM alleviated *E. coli* damage to poultry. In the current study, we investigated the effects of PHM on antioxidant capability, immune response, intestinal health, and gut microbiota composition of chicks infected with *E. coli* O78. We therefore aimed to further explore the role of the interaction between gut microbiota and antioxidant, anti-inflammatory, and anti-apoptotic in the process of PHM attenuating *E. coli* injury.

## Materials and methods

### Preparation and composition of polyherbal mixture

The PHM was prepared according to a previously described method (Liu et al., 2023). PHM is composed of 5 TCMs, including *Portulaca oleracea L., Radix Sophorae Flavescentis, Thalictrum glandulosissimum, Terra flava usta*, and *Pogostemon cablin*, in a 1:1:1:1:1 ratio. Then dry, crush, and sieve through an 80-mesh sieve to obtain the final PHM powder. The effective active ingredients and nutritive composition of PHM powder were listed in our previous research (Liu et al., 2023).

### Animals and treatments

All experimental protocols were approved by the Animal Care and Use Committee of the South China Agricultural University (approval number: SYXK 2022–0136, Guangzhou, China). One hundred and eighty 1-day-old chicks (Guangming No.2 white-feathered broiler, bred jointly by the Beijing Institute of Animal Husbandry and Veterinary Sciences of the Chinese Academy of Agricultural Sciences and Foshan Xanguang Farming Co., LTD.) were randomly divided into the CON group (basal diet), *E. coli* group (basal diet), PHM2 group (basal diet supplemented with 2 g/kg PHM), and PHM4 group (basal diet supplemented with 4 g/kg PHM), with 45 chicks per group in three cages, and were given free access to food and water. The *E. coli* O78 (repository number: CVCC1569; China Veterinary Microorganism Strains Preservation Management Center, Beijing, China) was obtained from cultivating it for 24 h at 37°C in Luria-Bertani (LB) broth (Difco, Sparks, USA). At the age of 17 days, except for the CON group, all the chicks were intraperitoneally injected with *E. coli* O78 (0.8 mL × 10^8^ CFU/mL), and the experiment was terminated after 48 h.

### Sample collection

48 h after infection with *E. coli*, a 5 mL blood sample was collected from the vein, and the chick was euthanized by cervical dislocation. Six sacrificed experimental chicks from each group were dissected, and the thymus, liver, spleen, and bursa of Fabricius were removed and weighed. Blood samples were centrifuged at 3000 × *g* for 20 min at 4°C, and serum was collected. A rapid separation of jejunum and ileum was performed, and a 0.5 cm sample of mid jejunum and ileum was placed in 4 % paraformaldehyde for intestinal morphometry. The ileal mucosa was scraped with a sterile scraper, and samples were collected into 2 mL cryotubes and flash-frozen in liquid nitrogen. Serum and frozen mucosa were stored at −80°C for further analysis.

### Production performance

Body weight (**BW**), body weight gain (**BWG**), and average daily gain (**ADG**) were analyzed at the end of the trial, along with the incidence of diarrhea and mortality after intraperitoneal injection of *E. coli*. The fatality rate of the chicks ((number of dead chicks/total number of chicks in each group) × 100 %) was calculated on the nineteenth day.

### Serum biochemical parameters

Serum biochemical indexes, including total protein (**TP**), albumin (**ALB**), alanine aminotransferase (**ALT**), aspartate aminotransferase (**AST**), and UREA, were determined via the biochemical analytical instrument PUZS-600B (Medical Equipment Co., Ltd., Beijing, China) using respective commercial assay kits (Shenzhen Mindray Biomedical Electronics Co., Ltd., Shenzhen, China).

### Determination of antioxidant enzyme activity and immunologic indices

The inflammation-related factors such as interleukin-6 (**IL-6**), interferon-γ (**IFN-γ**), interleukin-10 (**IL-10**), interleukin-1β (**IL-1β**), tumor necrosis factor-α (**TNF-α**), and caspase 8 were measured in chick serum using the corresponding kit according to the instruction manual of the kit manufacturer (Shanghai Coibo Bio Technology Co., Ltd., Shanghai, China). Serum levels of three factors related to intestinal barrier function: diamine oxidase (**DAO**), lipopolysaccharide **(LPS**), and D-lactate (**D-LA**) were also measured. Jejunum and ileum were homogenized in ice-cold phosphate-buffered saline at a ratio of 1:10 (g/mL) and centrifuged at 3000 × *g*, 4°C for 10 min.

### Histopathological analysis of jejunum and ileum

According to the previous method (Liu et al., 2023), the jejunal and ileal tissues were collected, washed with PBS, fixed with 4 % paraformaldehyde solution, dehydrated with ethanol, embedded in paraffin, sliced, and finally stained with hematoxylin and eosin (**H&E**).

### Tissue RNA extraction and analysis of relative expression levels of genes

Total RNA was extracted from cecal mucosa (50∼100 mg) using a Trizol kit (Invitrogen, Carlsbad), and NanoDrop Lite (ThermoFisher Scientific, Waltham) was employed to measure the RNA concentration. Thereafter, 1 μg of the RNA sample was used to synthesize cDNA using a reverse transcription kit (Vazyme, Nanjing, China). Real-time fluorescence quantitative PCR (**qRT-PCR**) analysis was conducted on a CFX96 contact real-time PCR detection system (Bio-Rad, Hercules) using a qRT-PCR kit (Vazyme, Nanjing, China), with GAPDH as the internal reference gene. The qRT-PCR premix contained quantitative primers and the cDNA template. The experiment was conducted in triplicate, and the expression level of the target gene was calculated using the delta-delta (2^−ΔΔCt^) method. Primers for genes related to antioxidants (CAT, SOD1, GSH-Px, Keap-1, Nrf2, and HO-1), immunity (IL-1β, IL-10, IL-6, iNOS, COX-2, TNF-α, TLR4, NF-κB, and MyD88), apoptosis (Bax, Bcl-2, Caspase 3, and Caspase 8), and intestinal barrier function (Claudin-1, Occludin, ZO-1, and Mucin-2) were designed using Primer Premier 6.0 software (Premier Biosoft International, United States) and synthesized by Tsingke Biotechnology Co., Ltd. (Beijing, China) (**Table S1**).

### 16S rRNA sequencing and gut microbiota analysis

The fresh cecal digest samples of *E. coli*-infected chicks in the 4 treatment groups (CON, *E. coli*, PHM2, and PHM4) were used to evaluate the cecal microflora community (*n* = 5). The cecal digesta microbiota genomic DNA 16S rRNA v3-v4 region was sequenced using a high-throughput sequencing method (Illumina NovaSeq 6000, 250 PE). Briefly, microbial genomic DNA was extracted from the samples using the E.Z.N.A. DNA Kit (Omega Bio-tek, Norcross, GA), according to the manufacturer's protocol. After the 16S rRNA gene v3-v4 region amplification, the resulting PCR products were extracted and purified. The purified amplicons were then pooled in equimolar amounts and subjected to paired-end sequencing on an Illumina MiSeq platform (Illumina, San Diego). The sequencing process and instruments were provided by Personalbio Technology CO., Ltd. (Shanghai, China). Microbiological data analysis, PICRUSt, and correlation analysis were performed using the software in the cloud platform of Shanghai Maggie Biomedical Technology Co., Ltd. (Shanghai, China). The repository names and accession numbers are listed below: https://www.ncbi.nlm.nih.gov/sra/PRJNA1203957.

### Statistical analysis

All data was normalized using SPSS software (version 19.0) and subjected to statistical analysis using one-way analysis of variance (ANOVA). Duncan's multiple comparison test was used to compare differences among the different groups. Data is expressed as the mean ± standard error of the mean (SE). Differences were considered significant at *P* < 0.05.

## Results

### Effects of PHM on body weight and mortality rates of chicks

**Fig. 1:**
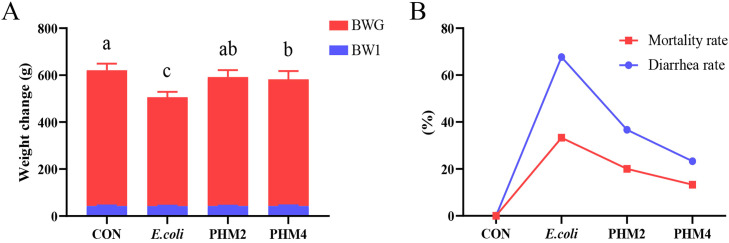
(A) Body-weight changes of chicks fed luteolin for 19 days. (B) The mortality rates (%) and diarrhea rates (%) of chicks in the control group, *E. coli* group, PHM2 (2 g/kg) group, and PHM4 (4 g/kg) group after *E. coli* injection. The data are expressed as mean ± SEM. A statistical difference (*P* < 0.05) was indicated using different letters. BMG, body weight gain. BW 1, 1-day-old weight.

The effect of PHM on clinical symptoms of *E. coli* infection in chicks, including body weight, mortality rate, diarrhea rate, etc., was monitored (Table 1, Fig. 1). At 19 days of age, which is the endpoint of the experiment, Escherichia coli caused a significant decrease in the weight of the chicks (*P* < 0.05), and the weight was increased by different doses of PHM in chicks induced by *E. coli* (*P* < 0.05). Meanwhile, PHM markedly reversed the ADG in *E. coli* induced chicks (*P* < 0.05) (Fig. 1A). BWG also obviously increased in *E. coli*-infected chick treatment with PHM (*P* < 0.05). In addition, the mortality and diarrhea rates of chicks infected with *E. coli* were reduced by PHM (Fig. 1**B**).

**Table 1:** The growth performance of chicks infected with *E. coli* and fed on PHM at 19 days.

Items	CON	*E. coli*	PHM2	PHM4	SEM	*P*-value
BW 1 (g)	42.87	42.57	42.75	42.83	0.383	0.994
BW 19 (g)	621.25^a^	537.92^c^	592.42^ab^	582.50^b^	8.542	0.001
ADG (g)	27.54^a^	23.59^c^	26.17^ab^	25.70^b^	0.404	0.001

^a-c^ Means within a row with no common superscript differ significantly (*P* < 0.05). Values are means (*n* = 30). Abbreviations: BW 1 and BW 19, body weight in 1-day-old and 19-days-old. ADG, average daily gain.

### Determination of serum biochemical indicators, antioxidant capacity and caspase 8

The levels of serum biochemical indicators, antioxidants, and caspase 8 were measured in serum and shown in Table 2. The levels of AST, T-AOC, and UREA in serum were not significantly altered, but the levels of ALB, TP, SOD, and GSH-Px were markedly reduced (*P* < 0.05), while ALT, MDA, and caspase 8 were markedly increased (*P* < 0.05) in the serum of chicks infected with *E. coli*. PHM significantly reversed (*P* < 0.05) the level of ALT, TP, SOD, GSH-Px, MDA, and caspase 8 and decreased UREA (*P* < 0.05) in *E. coli*-infected chicks, and the effect of the PHM4 group was better than that of the PHM2 group.

**Table 2:** Serum biochemical indices and antioxidant enzyme activity of *E. coli*-infected chicks fed diets supplemented with PHM.

Items	CON	*E. coli*	PHM2	PHM4	SEM	*P*-value
AST (U/L)	266.60	286.60	279.40	281.16	3.675	0.275
ALT (U/L)	4.20^c^	6.60^a^	5.5^ab^	5.12^bc^	0.282	0.010
ALB (g/L)	11.06^a^	9.04^b^	9.24^b^	9.72^ab^	0.277	0.027
TP (g/L)	29.52^a^	25.20^b^	28.00^a^	28.04^a^	0.488	0.005
UREA (mmol/L)	1.48^ab^	1.78^a^	1.42^b^	1.02^c^	0.080	0.002
SOD (U/mL)	154.84^a^	121.49^c^	123.49^bc^	149.68^ab^	5.297	0.029
GSH-Px (U/mL)	58.04^a^	36.85^c^	41.36^bc^	50.03^ab^	2.454	0.003
T-AOC (U/mL)	0.78	0.72	0.79	0.75	0.021	0.692
MDA (nmol/mL)	1.89^b^	2.73^a^	2.26^ab^	1.96^b^	0.122	0.027
Caspase 8 (pmol/L)	137.59^b^	159.34^a^	130.27^b^	133.07^b^	3.467	0.003

^a-c^ Means within a row with no common superscript differ significantly (*P* < 0.05). Values are means (*n* = 6). Abbreviations: CON, control; *E. coli, Escherichia coli*; PHM, polyherbal mixture; AST, aspartate aminotransferase; ALT, alanine transaminase; ALB, albumin; TP, total protein; SOD, superoxide dismutase; GSH-Px, glutathione peroxidase; T-AOC, total antioxidant capacity; MDA, malondialdehyde.

### Organ indices and determination of SIgA and serum immunoglobulins and cytokines

The effects of PHM on the immune function of chicks infected with *E. coli*, including organ index, immunoglobulin, and cytokines, were also tested (Table 3). The liver index (*P* < 0.05) and spleen index (*P* < 0.05) in *E. coli*-infected chicks were markedly higher than that in the CON group. PHM treatment markedly reduced liver index (*P* < 0.05) and spleen index (*P* < 0.05) and raised bursa index (*P* < 0.05) in *E. coli*-infected chicks. After infecting chicks with *Escherichia coli*, the levels of IgA, IgG, and IL-10 in the serum were significantly reduced (*P* < 0.05), and the levels of TNF-α and IFN-γ in the serum were significantly increased (*P* < 0.05). Meanwhile, after supplementing with PHM, the concentrations of IgA, IgG, IL-10, TNF-α, and IFN-γ in the serum were reversed (*P* < 0.05). In addition, the concentrations of sIgA (*P* < 0.05) in the jejunum and ileum were also reduced in *E. coli*-infected chicks and increased by PHM supplementation.

**Table 3:** Immune-related indices of *E. coli*-infected chicks fed diets supplemented with PHM.

Items	CON	*E. coli*	PHM2	PHM4	SEM	*P*-value
Liver index (%)	2.70^b^	4.56^a^	2.80^b^	2.63^b^	0.215	< 0.001
Spleen index (%)	0.08^b^	0.18^a^	0.11^b^	0.10^b^	0.014	0.037
Bursa index (%)	0.18^ab^	0.15^b^	0.22^a^	0.23^a^	0.011	0.010
Thymus index (%)	0.33	0.23	0.32	0.28	0.015	0.075
IgA (μg/mL)	212.38^b^	170.36^c^	213.88^b^	256.56^a^	9.213	0.003
IgG (μg/mL)	354.67^b^	360.44^b^	378.03^ab^	427.93^a^	10.431	0.037
IgM (μg/mL)	851.80	836.60	832.80	847.20	9.936	0.913
IL-1β (pg/mL)	274.89	307.89	282.44	299.67	6.266	0.226
IL-10 (pg/mL)	47.79^b^	39.92^c^	46.46^b^	51.33^a^	1.078	< 0.001
TNF-α (pg/mL)	45.75^b^	52.90^a^	38.65^c^	37.12^c^	1.619	< 0.001
IFN-γ (pg/mL)	47.97^b^	56.86^a^	40.92^c^	45.03^b^	1.471	< 0.001
Jejunal sIgA (mg/g protein)	6.35^a^	5.37^b^	6.12^a^	6.02^a^	0.101	< 0.001
Ileal sIgA (mg/g protein)	6.06^a^	5.53^b^	5.88^ab^	6.18^a^	0.081	0.012

^a-c^ Means within a row with no common superscript differ significantly (*P* < 0.05). Values are means (*n* = 6). Abbreviations: CON, control; *E. coli, Escherichia coli*; PHM, polyherbal mixture; IgA, immunoglobulin A; IgG, immunoglobulin G; IgG, immunoglobulin M; IL-1β, interleukin-1β; IL-10, interleukin-10; TNF-α, tumor necrosis factor-α; IFN-γ, interferon-γ; sIgA, secreted immunoglobulin A.

### Effect of PHM on the mRNA expression of antioxidant-related genes in E. coli-infected chicks

**Fig. 2:**
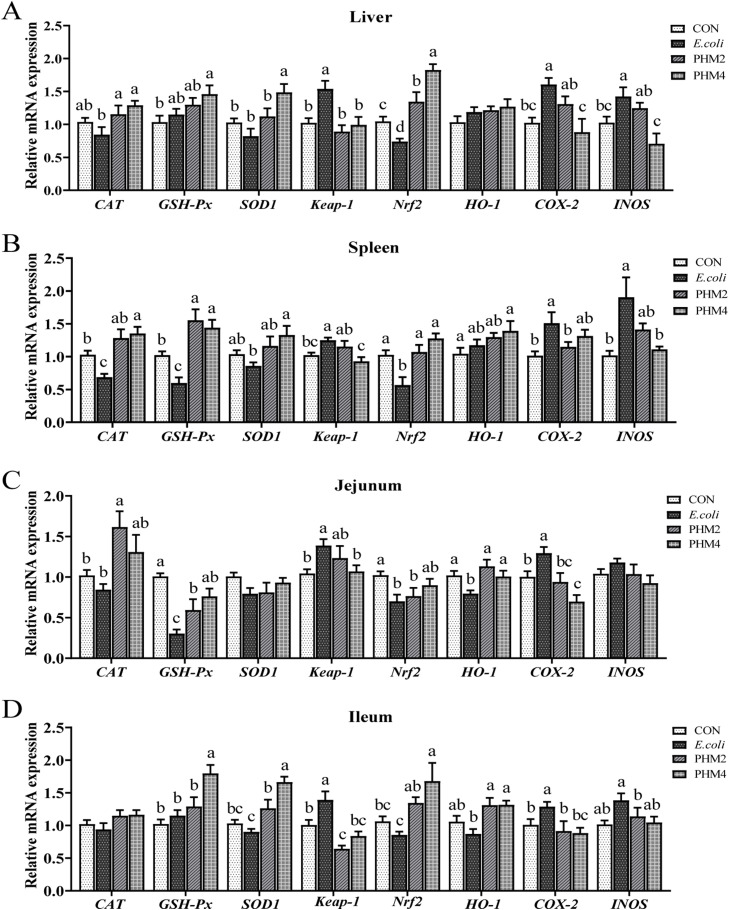
PHM effects on antioxidant-related gene expression in *E. coli*-infected chicks. (A-D) The expression levels of antioxidant-related genes (CAT, GSH-Px, SOD1, Keap-1, Nrf2, HO-1, COX-2, and INOS) in the liver, spleen, jejunum, and ileum, respectively. Abbreviations: CON, control; *E. coli, Escherichia coli*; PHM, polyherbal mixture; CAT, catalase; SOD1, copper and zinc superoxide dismutase; GSH-Px, Glutathione peroxidase; Keap-1, Kelch-like ECH-associated protein 1; Nrf2, nuclear factor E2-related factor 2; HO-1, heme oxygenase-1. Data are presented as means ± SEM (*n* = 6). Different letters indicate significant differences in the interaction effect (*P* < 0.05).

The relative mRNA expression of antioxidant-related genes was presented in Fig. 2. Compared with the CON group, the mRNA expression of CAT, GSH-Px, and Nrf2 was reduced (*P* < 0.05) and Keap-1, COX-2, and INOS were increased (*P* < 0.05) in the spleen of the *E. coli*-infected chicks. Unlike the results in the spleen, the expression of CAT and GSH-Px in the liver, CAT and INOS in the jejunum, and CAT, GSH-Px, Nrf2, and INOS in the ileum were not altered by *E. coli*, but the expression of HO-1 (*P* < 0.05) in the jejunum was obviously reduced by *E. coli*. The supplementation of PHM significantly restored the changes in antioxidant-related genes caused by *E. coli* in the liver, spleen, jejunum, and ileum of chicks (*P* < 0.05).

### Effect of PHM on the mRNA expression of inflammation-related genes in E. coli-infected chicks

**Fig. 3:**
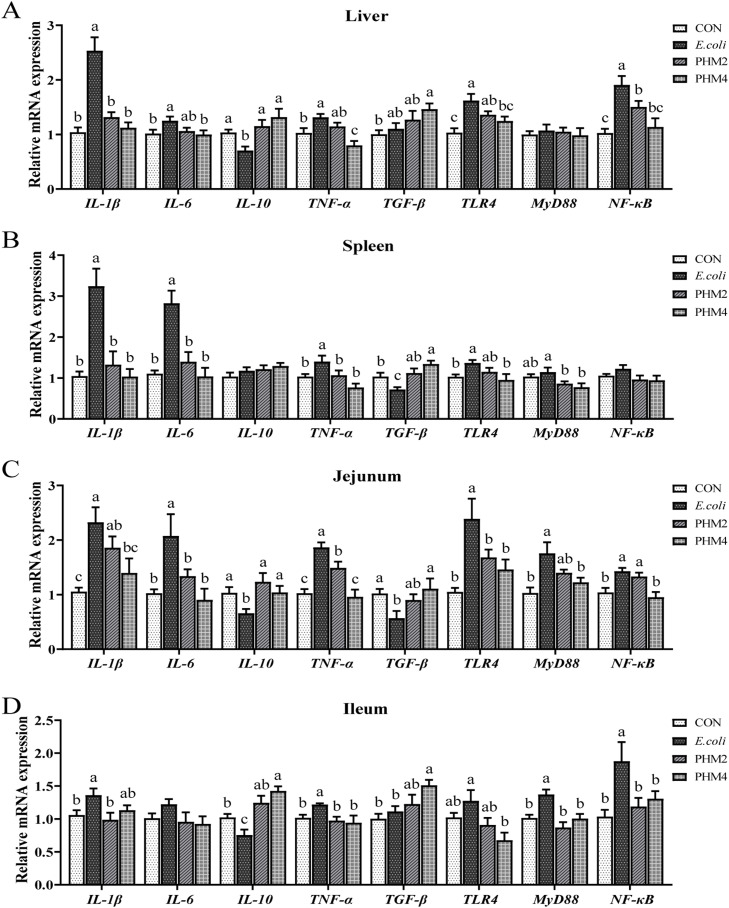
PHM effects on inflammation-related gene expression in *E. coli*-infected chicks. (A-D) The expression levels of immune-related genes (IL-1β, IL-10, IL-6, INOS, COX-2, TNF-α, TLR4, NF-κB, and MyD88) in the liver, spleen, jejunum, and ileum, respectively. Abbreviations: CON, control; *E. coli, Escherichia coli*; PHM, polyherbal mixture; IL-1β, interleukin-1β; IL-10, interleukin-10; IL-6, interleukin-6; INOS, inducible nitric oxide sythase; COX-2, cyclooxygenase-2; TNF-α, tumor necrosis factor-α; TLR4, toll like receptor 4; NF-κB, nuclear factor kappa B; MyD88, myeloid differentiation factor 88. Data are presented as means ± SEM (*n* = 6). Different letters indicate significant differences in the interaction effect (*P* < 0.05).

The mRNA expression of inflammation-related genes was also observed in chicks (Fig. 3). The expression of IL-1β and TNF-α in the liver, spleen, jejunum, and ileum (*P* < 0.05); IL-6 and TLR-4 in the liver, spleen, and jejunum (*P* < 0.05); TGF-β in the spleen and jejunum (*P* < 0.05); MyD88 in the jejunum and ileum (*P* < 0.05); IL-10 and NF-κB in the liver, jejunum, and ileum showed significant differences between the CON group and *E. coli* groups (*P* < 0.05). However, PHM treatment markedly reduced the expression of IL-1β, IL-6, TNF-α, TIR4, MyD88, and NF-κB and increased IL-10 and TGF-β in the liver, spleen, jejunum, and ileum of *E. coli*-infected chicks (*P* < 0.05).

### Effect of PHM on the mRNA expression of apoptosis-related genes in E. coli-infected chicks

**Fig. 4:**
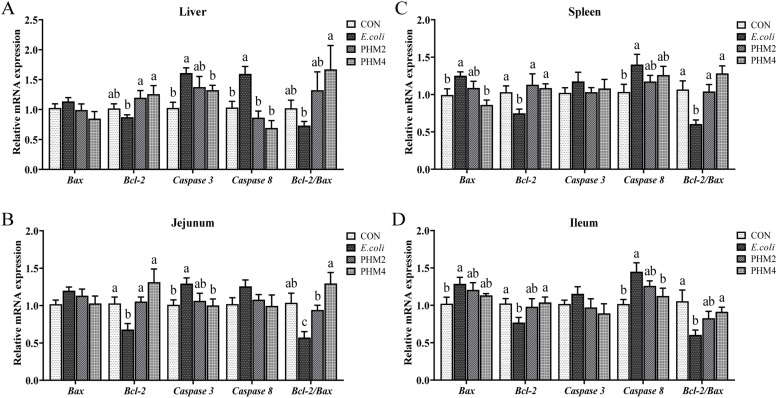
PHM effects on anti-apoptotic ability in *E. coli*-infected chicks. (A-D) The expression levels of apoptosis-related genes (Bax, Bcl-2, Caspase 3, and Caspase 8) and the ratio of Bax/Bcl-2 in the liver, spleen, jejunum, and ileum, respectively. Abbreviations: CON, control; E. coli, *Escherichia coli*; PHM, polyherbal mixture; Bax, Bcl-2-associated X protein; Bcl-2, B-cell lymphoma-2. Data are presented as means ± SD (*n* = 6). Different letters indicate significant differences in the interaction effect (*P* < 0.05).

The results of apoptosis-related genes were shown in Fig. 4. Compared with the CON group, chick infection with *E. coli* increased the expression of Bax (*P* < 0.05) in the spleen and ileum, caspase 3 (*P* < 0.05) in the liver and jejunum, and caspase 8 (*P* < 0.05) in the liver, spleen, and ileum; decreased the expression of Bcl2 (*P* < 0.05) in the liver, spleen, jejunum, and ileum; and decreased the ratio of Bcl2/Bax (*P* < 0.05) in the spleen, jejunum, and ileum. PHM significantly restored the expression of Bax, Bcl2, Caspase 3, and Caspase 8 and the ratio of Bcl2/Bax in *E. coli*-infected chicks (*P* < 0.05).

### Effect of PHM on Intestinal permeability biomarkers and barrier function in E. coli-infected chicks

**Fig. 5:**
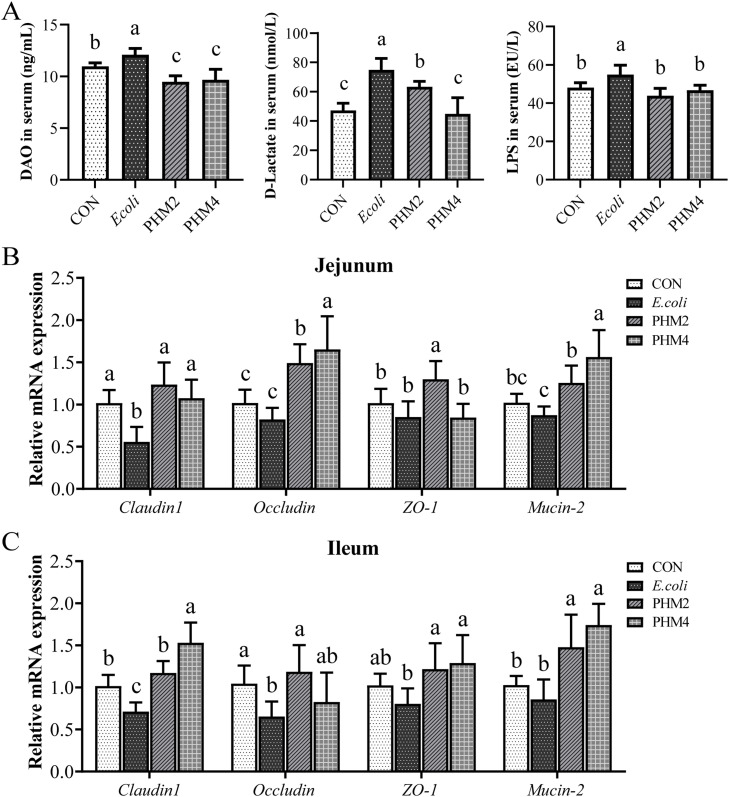
Effects of dietary PHM supplementation on intestinal permeability biomarkers and barrier function of *E. coli*-infected chicks. (A) The concentration of DAO, d-lactate, and LPS in serum. (B-C) The mRNA expression of intestinal tight junction proteins (Claudin-1, Occludin, and ZO-1) and Mucin-2 in jejunum and ileum. Abbreviations: CON, control; *E. coli, Escherichia coli*; PHM, polyherbal mixture; DAO, diamine oxidase; LPS, lipopolysaccharide; ZO-1, zonula occludens-1. Data are presented as means ± SEM (*n* = 6). Different letters indicate significant differences in the interaction effect (*P* < 0.05).

Intestinal permeability was assessed by measuring the levels of D-lactate, DAO, and LPS in plasma (Fig. 5). Compared with the CON group, the concentration of D-lactate, DAO, and LPS in the serum of the *E. coli* group was significantly increased. Supplementing PHM markedly decreased the levels of D-lactate, DAO, and LPS in the serum. The H&E staining (**Fig. S1**) showed a significant difference in intestinal morphology among the four groups. Compared with the CON group, the *E. coli* group showed significant pathological changes in the jejunal and ileal morphology, including epithelial cell loss and destruction of the villi structure. Compared with the *E. coli* group, the PHM group obviously decreased the villi structural damage in the jejunum and ileum of broilers infected with APEC. Meanwhile, the barrier function in the jejunum and ileum was also analyzed in Fig. 5. The expression of intestinal barrier-related genes, including claudin-1 (*P* < 0.05) and occludin (*P* < 0.05) in the jejunum and ileum and occludin (*P* < 0.05) in the ileum, was significantly lower in the *E. coli* group than that in the CON group, whereas it was significantly higher in the PHM group than that in the *E. coli* group. Meanwhile, PHM significantly upregulated the expression of ZO-1 (*P* < 0.05) and Mucin-2 (*P* < 0.05), which were not significantly reduced by *E. coli*.

### Microbial diversity of the cecal digesta in E. coli-infected chicks

**Fig. 6:**
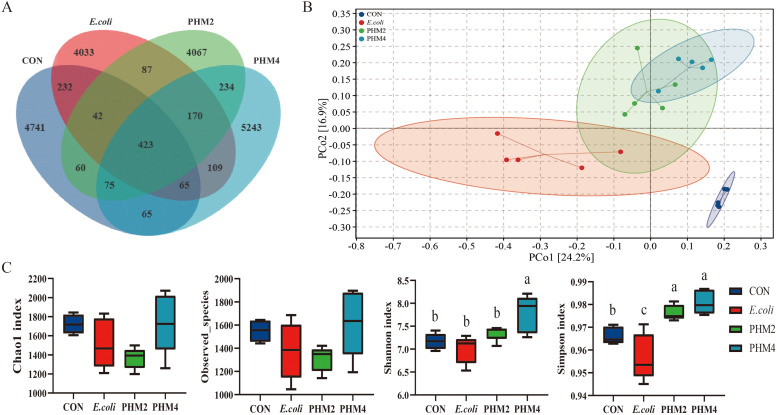
Effects of dietary PHM supplementation on the microbial diversity in the cecal contents of *E. coli*-infected chicks. (A) Venn diagram of observed taxonomic unit (OTU) levels in cecal contents. (B) β diversity was shown as PCoA analysis. (C) Alpha diversity at the OTU level. Abbreviations: CON, control; *E. coli, Escherichia coli*; PHM, polyherbal mixture. Data are presented as means ± SEM (*n* = 5). Different letters indicate significant differences in the interaction effect (*P* < 0.05).

To explore the role of intestinal microbiota in *E. coli*-infected chicks with dietary supplementation of PHM, we profiled the composition of intestinal microbiota in all groups using 16S rRNA gene high-throughput sequencing. The effects of PHM supplementation on microbial diversity in the cecal contents of *E. coli*-infected chicks are shown in Fig. 6. The Venn diagram results showed that there were 5703 OTUs in the CON group, 5161 OTUs in the *E. coli* group, 5158 OTUs in the PHM2 group, and 6375 OTUs in the PHM4 group. Among them, there were a total of 762 OTUs in the CON group and *E. coli* group, 722 and 767 OTUs in *E. coli* group and PHM2 and PHM4 groups, and 902 OTUs in PHM2 and PHM4 groups, and 423 OTUs in these four groups (Fig. 6**A**). The plot of PCoA showed significant differences between the CON group and *E. coli* group, while the *E. coli* group and PHM2 group showed similar distribution trends, but the PCoA distribution in PHM4 showed significant changes (Fig. 6**B**). The alpha diversity index, including the Chao1 index, Observed-species, Shannon index (*P* < 0.05), and Simpson index (*P* < 0.05), was significantly reduced by *E. coli*, while the supplementation of PHM increased the levels of these indices, especially PHM4 (Fig. 6**C**).

### Microbial composition of the cecal digesta in E. coli-infected chicks

**Fig. 7:**
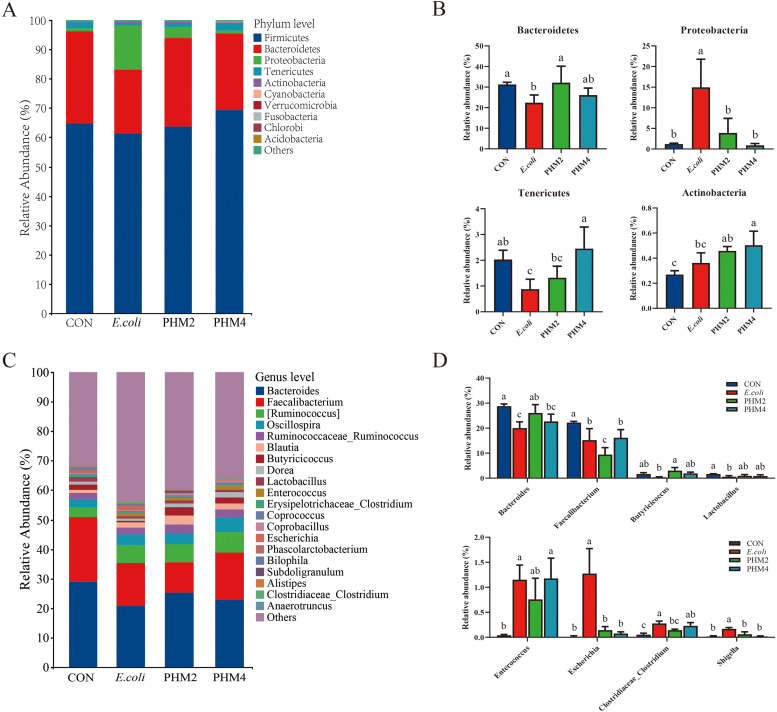
Effects of PHM2 group on the microbial composition in the cecal contents of *E. coli*-infected chicks. The relative abundance of gut bacteria at the (A) phylum and (C) genus level in different treatments. Analysis of the significant differences in microbiota composition on the (B) phylum and (D) genus level. Abbreviations: CON, control; *E. coli, Escherichia coli*; PHM, polyherbal mixture. Data are presented as means ± SEM (*n* = 5). Different letters indicate significant differences in the interaction effect (*P* < 0.05).

**Fig. 8:**
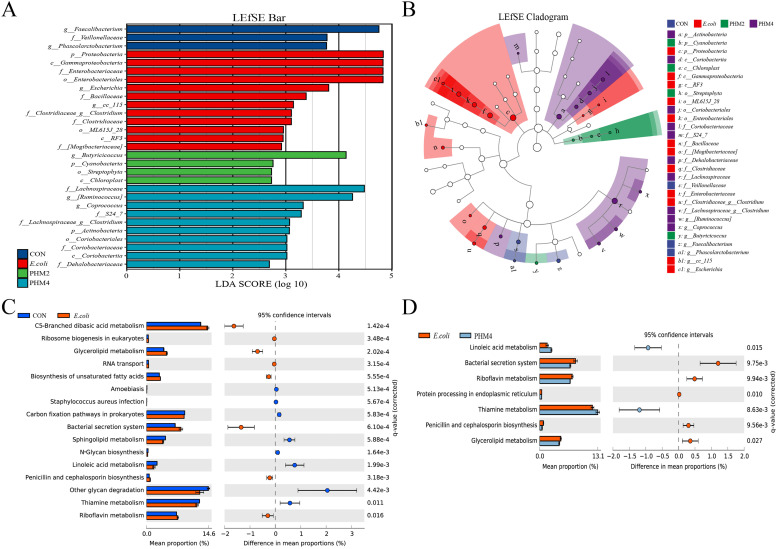
Changes and functional prediction of cecal microflora in broilers infected with APEC after addition of PHM. (A, B) LEfSe analysis and branching diagram of the evolution scatter plot. (C, D) Analysis of functional prediction between the three groups. Abbreviations: CON, control; *E. coli, Escherichia coli*; PHM, polyherbal mixture. * represents a significant difference (*P* < 0.05), ** represents a significant difference (*P* < 0.01).

To look for species with significant differences in abundance between taxa and species with significant differences between taxa at different levels, we compared the microbial communities of the four experimental groups by LEfSe analysis, and the results are shown in Fig. 7. At the phylum levels, *Firmicutes, Bacteroidetes*, and *Proteobacteria* were dominant, while at the genus level, *Bacteroidetes, Faecalibacterium*, and *Ruminococcus* dominated in abundance (*P* < 0.05) (Fig. 7**A, C**). Chicks infected with *E. coli* showed a reduction in the relative abundance of *Bacteroidetes* and *Tenericutes* and an increase in *Proteobacteria*, while chicks supplemented with PHM reversed the relative abundance of *Bacteroidetes, Proteobacteria*, and *Tenericutes* (*P* < 0.05) (Fig. 7**B**). At the genus level, the relative abundance of *Bacteroides, Faecalibacterium, Butyricicoccus, Lactobacillus,* and *Lachnospiraceae-Clostridium* was markedly decreased, and *Enterococcus, Escherichia,* and *Shigella* were significantly increased in cecal digesta by *E. coli* (*P* < 0.05) (Fig. 7**D**). However, dietary supplementation of PHM restored these changes (*P* < 0.05) (Fig. 7**D**). Finally, a linear discriminant analysis (LDA) effect size (LEfSe) analysis was conducted to further identify the enriched microbial community from phylum to genus among four groups with an LDA score >2.5 (Fig. 8**A, B**). The results of LEfSe analysis showed that some bacterial groups, including the genera *Faecalibacterium* in the CON group, had a higher score; the phylum *Proteobacteria*, the class *Gammaproteobacteria*, the family *Enterobacteriaceae*, the order *Enterobacteriales*, and the genera *Escherichia* in the *E. coli* group had a higher score, while some other bacterial groups, such as the genera *Butyricicoccus* in the PHM2 group and the family *Lachnospiraceae* and the genera *Ruminococcus* in the PHM4 group, had a higher score.

### Functional prediction of cecal microbiota

Phylogenetic Investigation of Communities by Reconstruction of Unobserved States (PICRUSt) was performed to analyze the possible levels of KEGG pathways (Fig. 8**C, D**). The prediction analysis identified 16 functional pathways between CON and *E. coli* groups and 7 functional pathways between *E. coli* and PHM4 groups. Among the 16 pathways identified, broilers infected with APEC notably increased the microbial gene abundance of membrane transport (bacterial secretion system), lipid metabolism (glycerolipid metabolism and biosynthesis of unsaturated fatty acids), and metabolism of cofactors and vitamins (riboflavin metabolism), while suppressing the microbial gene abundance of energy metabolism (carbon fixation pathways in prokaryotes), glycan biosynthesis and metabolism (N-glycan biosynthesis and other glycan degradation), lipid metabolism (sphingolipid metabolism and linoleic acid metabolism), and metabolism of cofactors and vitamins (thiamine metabolism). The dietary inclusion of PHM4 led to a significant increase in the microbial gene abundance related to the metabolism of cofactors and vitamins (thiamine metabolism) and lipid metabolism (linoleic acid metabolism) across all three levels of the KEGG pathways compared to the E. coli group. Conversely, the PHM4 group resulted in a decrease in the gene abundance associated with membrane transport (bacterial secretion system), lipid metabolism (glycerolipid metabolism), and metabolism of cofactors and vitamins (riboflavin metabolism).

### Analysis of key bacterial genera altered by PHM in E. coli-infected chicks

**Fig. 9:**
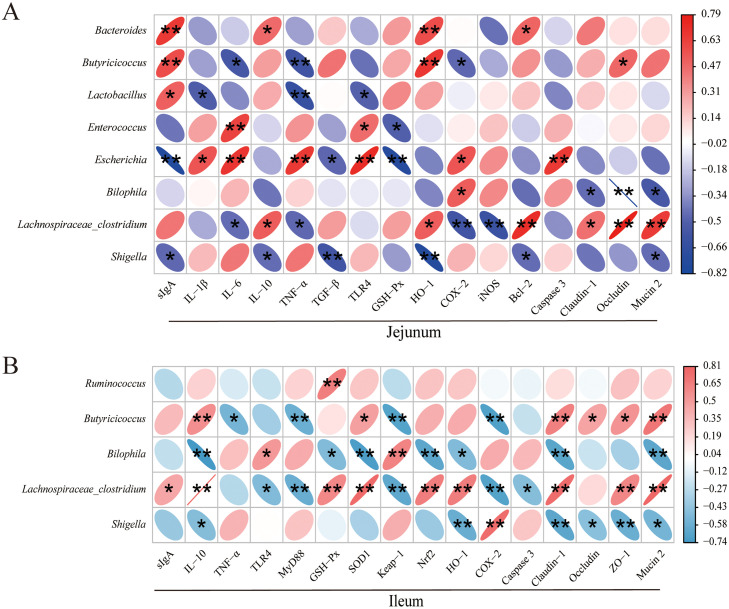
Correlation of intestinal microbiota with APEC-affected measurements of PHM. (A, B) The dominant differential microbials in the gut were related to the immune indexes, antioxidant-related genes, anti-apoptosis-related genes, and barrier function genes of the jejunum and ileum, respectively. * was judged as a trend with a difference at *P* < 0.05, ** represents a significant difference at *P* < 0.01 (*n* = 5).

To further explore the relationship between cecum microbiota and PHM treatment of *E. coli* in chicks, we conducted correlation analysis on changes between cecum microbiota and intestinal anti-inflammatory, antioxidant, and barrier-related functions treated with PHM. Correlation analysis showed stronger correlations between key gut bacterial genera and barrier, anti-inflammatory, and antioxidant functions of the jejunum, whereas *Escherichia* and *Lachnospiraceae-clostridium* were more strongly associated with the jejunum, and *Butyricicoccus, Bilophila*, and *Lachnospiraceae-Clostridium* were more strongly associated with the ileum (Fig. 9). And *Butyricicoccus, Bilophila, Lachnospiraceae-clostridium*, and *Shigella* were the shared key gut bacterial genera associated with the ileum and jejunum. More importantly, the regulation of PHM on the jejunum was positively correlated with the abundance of *Lachnospiraceae-clostridium* and *Butyricicoccus* and negatively correlated with *Escherichia, Bilophila* and *Shigella* (Fig. 9**A**), while PHM was positively correlated with *Butyricicoccus* and *Lachnospiraceae-clostridium* and negatively correlated with *Bilophila* and *Shigella* in the ileum (Fig. 9**B**). Taken together, these significantly correlated gut microbiota may be the key to improving the anti-inflammatory, antioxidant, and intestinal barrier effects of PHM in chicks infected with *E. coli*.

## Discussion

Pathogenic *E. coli* is one of the important challenges faced by the broiler farming industry, which seriously inhibits growth performance, reduces immune function and antioxidant capacity, exacerbates intestinal inflammation, and leads to cross-infection of various diseases, reducing economic benefits. Our previous study showed that PHM improved the growth performance, antioxidant capacity, and immune function of broilers (Liu et al., 2023). However, the effect of PHM on *E. coli*-infected broilers is still unclear. Therefore, in this study, we further investigated whether PHM could also improve the increase in inflammation, decrease in antioxidant capacity, and immunosuppression caused by *E. coli* in broilers.

In the present study, our results showed that PHM significantly inhibited the weight loss, mortality, and diarrhea rate caused by *E. coli*. Consistent with our findings, He et al. and Meng et al. showed that *E. coli* also caused weight loss and increased mortality, which were significantly reversed by the Chinese herbal compound (He et al., 2014; Meng et al., 2024). Consistent with previous studies, chickens infected with *E. coli* showed elevated spleen and liver organ indexes and abnormal blood biochemical markers related to liver function (Galal et al., 2018; Meng et al., 2024). The addition of PHM to the diet significantly improved the changes caused by *E. coli*. Immunosuppression is a common disaster in broiler breeding, which is induced by a variety of factors, and *E. coli* infection also induces and aggravates immunosuppression (Awad et al., 2019). In the present study, PHM significantly improved the concentration of immunoglobulin in serum and up-regulated the content of sIgA in jejunum and ileum, indicating that PHM significantly restored the immunosuppression caused by *E. coli*. Although it has not been reported that PHM enhances immunity, many studies have shown that *Sophora flavescens,* the Chinese herbal medicine in PHM, regulates immunity (Zhou et al., 2021).

Oxidative stress, TLR4/Myd88/NF-κB related inflammatory signaling, and apoptosis are important body responses in response to bacterial infection. Early supplementation of PHM in chickens infected with *E. coli* significantly inhibited inflammatory response, reduced oxidative stress, and reduced cell apoptosis, thereby alleviating the damage caused by *E. coli*. This is consistent with reports that *Portolaca oleracea L.* and *Sophora flavescens,* which are included in PHM, reduce inflammation levels and enhance antioxidant capacity (Wu et al., 2012; Lv et al., 2022; Zhang et al., 2024). Meanwhile, flavonoids and alkaloids from *Portolaca oleracea L.* and *Sophora flavescens* have also been shown to have anti-inflammatory and anti-oxidative effects (He et al., 2015; Yoon et al., 2019; Wang et al., 2024). The intestinal barrier, which is composed of intestinal epithelial cells and junctional complexes, is the first line of defense against pathogen invasion (Chen et al., 2021). The destruction of the intestinal barrier increases intestinal permeability, which increases the possibility of invasion by commensal bacteria and antigens, resulting in more inflammation and immunosuppression (Chelakkot et al., 2018; Zhang et al., 2022). Then, DAO, D-LA, and LPS are released into the blood after damage to the intestinal mucosa (Chen et al., 2017). Consistent with this, the present study showed that *E. coli* disrupted intestinal barrier integrity and increased serum DAO, D-LA, and LPS concentrations in chicks. However, PHM enhances intestinal barrier integrity and reduces intestinal permeability. These results suggested that PHM maintained intestinal barrier integrity disrupted by *E. coli* in chicks. Consistent with this, studies have reported that other formulations containing *Sophora flavescens* significantly repair the intestinal barrier (Wu et al., 2021).

Chicken gut microbiota plays an important role in the occurrence and development of diseases. When the gut is destroyed, opportunistic pathogens will proliferate rapidly and further destroy the homeostasis of intestinal flora (Rangan and Hang, 2017). Therefore, to further investigate the in-depth reasons why PHM attenuates *E. coli* damage, the regulation of intestinal flora by PHM was observed. The abundance of Bacteroidetes, which degrades high molecular weight compounds in the intestine to help the host obtain nutrients from the diet and promote normal gastrointestinal development, was significantly restored by PHM (Tremaroli and Backhed, 2012; Chen et al., 2018). In the present study, the abundance of *Proteobacteria* increased significantly after *E. coli* infection, which is consistent with previous reports that *Proteobacteria* are a marker of gut microbiota imbalance and that PHM significantly reduced its abundance (Shin et al., 2015). At the genus level, the abundance of *Faecalibacterium*, a butyrate producer and promoter of spermidine production (Zhu et al., 2024), and *Bacteroides, Butyricicoccus*, and *Lactobacillus*, which produce butyrate to reduce inflammation, were significantly reduced by *E. coli* (Shi et al., 2019; Li et al., 2021; Fan et al., 2023). *Enterococcus*, the natural inhabitants of the gastrointestinal tract, are known as opportunistic pathogens causing severe infections, and *Escherichia* and *Shigella*, the main harmful bacteria in the gut, were enriched in the cecum of chicks infected with *E. coli* (Zhang et al., 2013; Dufossé et al., 2023; Ben et al., 2024). The results of Spearman correlation analysis showed that the key differential bacteria, including *Butyricicoccus, Bilophila, Lachnospiraceae-clostridium, Shigella, etc.*, were closely related to the antioxidant, anti-inflammatory, and intestinal barrier function altered by PHM. The abundance of the beneficial bacteria *Butyricicoccus* and *Lachnospiraceae-clostridium*, which have been shown to produce SCFA, was significantly positively correlated with PHM improvem*ent* in *E. coli-infected chicks* (Sorbara et al., 2020)*. Bilophila* has been shown to exert deleterious effects on the gut and exacerbate intestinal inflammation, while *Bilophila wadsworthia*, belonging to the genus *Bilophila*, is associated with low degrees of systemic inflammatory disease (Salvado et al., 2024; Gan et al., 2025). Collectively, the effects of PHM on reducing inflammation, enhancing antioxidant capacity and immune function, and restoring intestinal barrier were positively correlated with the abundance of beneficial bacteria and negatively correlated with the abundance of harmful bacteria. Therefore, PHM treats diseases by reducing harmful bacteria in the gut and increasing beneficial bacteria to protect the intestinal barrier and reduce inflammation. This is consistent with previous studies on adjusting the composition of gut microbiota to treat diseases (Ahmed and Al-Massri, 2022; Cai and Kang, 2023). Similarly, gut microbiota also stimulated the production of metabolites by TCM to exert more important biological functions. Oxyberberine, the intestinal secondary metabolite of *Sophonae flavescens*, alleviates inflammation and maintains intestinal barrier integrity by inhibiting the NF-κB pathway (Li et al., 2020). Therefore, gut microbiota and TCM also have a mutually beneficial relationship, synergistically regulating intestinal homeostasis. KEGG analysis showed that the improvement of *E. coli*-infected chicks by PHM were related to the reversal of metabolic pathways cofactors and vitamins (thiamine metabolism and riboflavin metabolism) and lipid metabolism (linoleic acid metabolism and glycerolipid metabolism), membrane transport (bacterial secretion system) through differential gut microbiota. Thiamine and riboflavin, whose metabolic pathways are significantly upregulated by PHM, are cofactors of hundreds of enzymes that are essential for cellular and mitochondrial energy metabolism (Takeda and Dai, 2024). The linoleic acid metabolism, as a key pathway for the production of omega-6 fatty acids, is a precursor of the known inflammatory mediator arachidonic acid (Hu et al., 2024). And glycerolipid metabolism was positively correlated with the peroxisomal pathway (Wang et al., 2022). In the present study, PHM significantly reduced the linoleic acid and glycerolipid metabolism, which may be the key to PHM reducing inflammation and enhancing antioxidant function. Bacterial pathogens use secretion systems to secrete effectors into the environment or directly into the cytoplasm of target cells to achieve pathogenicity or survival (Wang et al., 2024). Bacterial pathogens, mainly Gram-negative bacteria, use bacterial secretion system to secrete effectors into the environment or directly into the cytoplasm of target cells to achieve pathogenicity or survival. The reduction of bacterial secretion system by PHM also led to a decrease in the abundance of pathogenic bacteria in the gut, especially Gram-negative bacteria, which was consistent with the reduction of *Bilophila* and *Shigella* in the gut by PHM. Taken together, these results indicated that the alleviation of PHM in chicks infected with *E. coli* was closely related to the gut microbiota, which is consistent with many reports (Lv et al., 2017; Ye et al., 2022).

## Conclusions

According to this study, our results showed that PHM significantly reversed the weight loss, diarrhea rate, and mortality in *E. coli*-infected chicks. PHM significantly enhanced the antioxidant capacity, repaired the immune response and intestinal barrier, and decreased proinflammatory cytokines and apoptosis-related genes in serum and different tissues, respectively. In addition, PHM also repaired intestinal injury to further reduce the serum concentrations of D-lactate, DAO, and LPS. PHM also significantly regulated the composition of cecal gut microbiota, especially the key phylotypes, which were significantly correlated with the improvement of PHM on *E. coli*-induced chicks. Among them, the antioxidant capacity, intestinal barrier function, and anti-inflammatory effects of PHM were significantly positively correlated with *Butyricicoccus, Lachnospiraceae-clostridium*, and significantly negatively correlated with *Bilophila* and *Shigella*. These findings provide evidence for PHM as a therapeutic strategy for *E. coli* diseases, but the underlying causes need to be further explored.

## Declaration of competing interest

The authors declare that they have no known competing financial interests or personal relationships that could have appeared to influence the work reported in this paper.

## Appendix Supplementary materials

Image, image 1

Image, application 2
