# Supplementary figures and images for: Modulatory effects of polyherbal mixture on the immuno-antioxidant capacity and intestinal health of chicks infected with Escherichia coli O78
Source: Poult Sci. 2025 Apr 12;104(6):105156. doi: 10.1016/j.psj.2025.105156 (PMC12032338; doi:10.1016/j.psj.2025.105156)

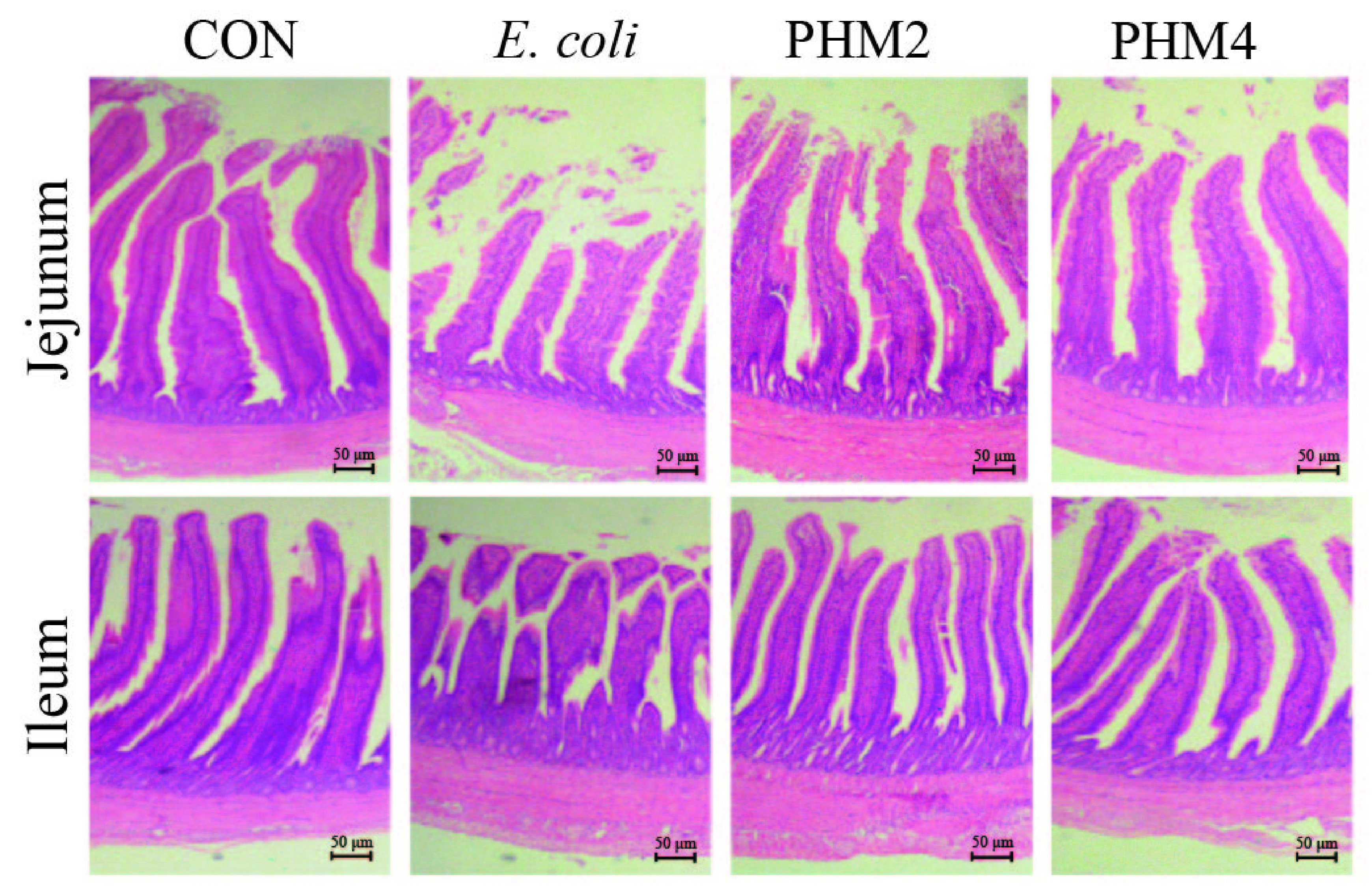

Supplement: Supplementary file 1 [file mmc1.jpg]
