# Supplementary material for: Modulatory effects of polyherbal mixture on the immuno-antioxidant capacity and intestinal health of chicks infected with Escherichia coli O78
Source: Poult Sci. 2025 Apr 12;104(6):105156. doi: 10.1016/j.psj.2025.105156 (PMC12032338; doi:10.1016/j.psj.2025.105156)
Supplement: Supplementary file 2 [file mmc2.docx]

**Supplementary information**

Table S1. The gene sequence for RT-qPCR

| Gene | Direction | Sequence (5′ - 3′) | Accession number |
| --- | --- | --- | --- |
| *CAT* | Forward | GCCACATGGTGACTACCCTC | NM_001031215.2 |
|  | Reverse | TGTTGCTAGGGTCATACGCC |  |
| *GSH-Px* | Forward | GACCAACCCGCAGTACATCA | NM_001277853.2 |
|  | Reverse | GAGGTGCGGGCTTTCCTTTA |  |
| *SOD1* | Forward | GAGCGGGCCAGTAAAGGTTA | NM_205064.155 |
|  | Reverse | CCCTTTGCAGTCACATTGCC |  |
| *Keap-1* | Forward | AGCAGCGTGGAGAGGTATGA | KU321503.1 |
|  | Reverse | GCGTACAGCAGTATGTTCAGC |  |
| *HO-1* | Forward | ACGAGTTCAAGCTGGTCACG | NM_205344.1 |
|  | Reverse | GGATGCTTCTTGCCAACGAC |  |
| *Nrf2* | Forward | CGCTTTCTTCAGGGGTAGCA | NM_205117.1 |
|  | Reverse | AGTTCGGTGCAGAAGAGGTG |  |
| *INOS* | Forward | CCTGGGTTTCAGAAGTGGC | NM_204961.1 |
|  | Reverse | CCTGGAGGTCCTGGAAGAGT |  |
| *COX-2* | Forward | TCCACCAACAGTGAAGGACA | M64990.1 |
|  | Reverse | GGACCAAGCCAAACACCTC |  |
| *TNF-α* | Forward | CCTACCCTGTCCCACAACCT | AF000631 |
|  | Reverse | TGAACTGGGCGGTCATAGAA |  |
| *IL-1β* | Forward | CAGCCTCAGCGAAGAGACCTT | NM_204524.2 |
|  | Reverse | ACTGTGGTGTGCTCAGAATCC |  |
| *IL-6* | Forward | AAATCCCTCCTCGCCAATCT | HM179640 |
|  | Reverse | CCCTCACGGTCTTCTCCATAAA |  |
| *IL-10* | Forward | ATCCAGAGACGATGAACTTAACA | AJ621614 |
|  | Reverse | TGCTTGATGGCTTTGCTCCT |  |
| *NF-κB* | Forward | GTGTGAAGAAACGGGAACTG | NM_205129 |
|  | Reverse | GGCACGGTTGTCATAGATGG |  |
| *TGF-β* | Forward | ATGTGTTCCGCTTTAACGTGTC | NM_205454.1 |
|  | Reverse | GCTGCTTTGCTATATGCTCATC |  |
| *TLR4* | Forward | CATCTCTGGAGTTCCTGCTGAA | NM_001030693.1 |
|  | Reverse | TGTATGGATGTGGCACCTTGA |  |
| *MyD88* | Forward | ATCCGGACACTAGAGGGAGG | NM_001030962.1 |
|  | Reverse | GGCAGAGCTCAGTGTCCATT |  |
| *Bax* | Forward | GGTGACAGGGATCGTCACAG | XM_422067 |
|  | Reverse | TAGGCCAGGAACAGGGTGAAG |  |
| *Bcl-2* | Forward | GCTGCTTTACTCTTGGGGGT | NM_205339.2 |
|  | Reverse | CTTCAGCACTATCTCGCGGT |  |
| *Caspase3* | Forward | TGCATGTCACTGAACTCCCC | AF083029.1 |
|  | Reverse | GCTTGGCAAACTTCTGGTGG |  |
| *Caspase8* | Forward | TAAAATGACCAGCCGACCCC | NM_204592.4 |
|  | Reverse | TCTGCATCCACATGTGTCCC |  |
| *ZO-1* | Forward | CCACCTCAGAATAAGCCAGCAAT | XM_015278981.2 |
|  | Reverse | CGGTTGTAAGGAGTGACTGTT |  |
| *Mucin-2* | Forward | AGGAATGGGCTGCAAGAGAC | XM_001234581.3 |
|  | Reverse | GTGACATCAGGGCACACAGA |  |
| *Claudin-1* | Forward | TGGCCACGTCATGGTATGG | NM_001013611.2 |
|  | Reverse | AACGGGTGTGAAAGGGTCATAG |  |
| *Occludin* | Forward | ACGGCAGCACCTACCTCAA | NM_205128.1 |
|  | Reverse | GGGCGAAGAAGCAGATGAG |  |
| *GAPDH* | Forward | GGTGGTGCTAAGCGTGTTAT | K01458 |
|  | Reverse | ACCTCTGTCATCTCTCCACA |  |


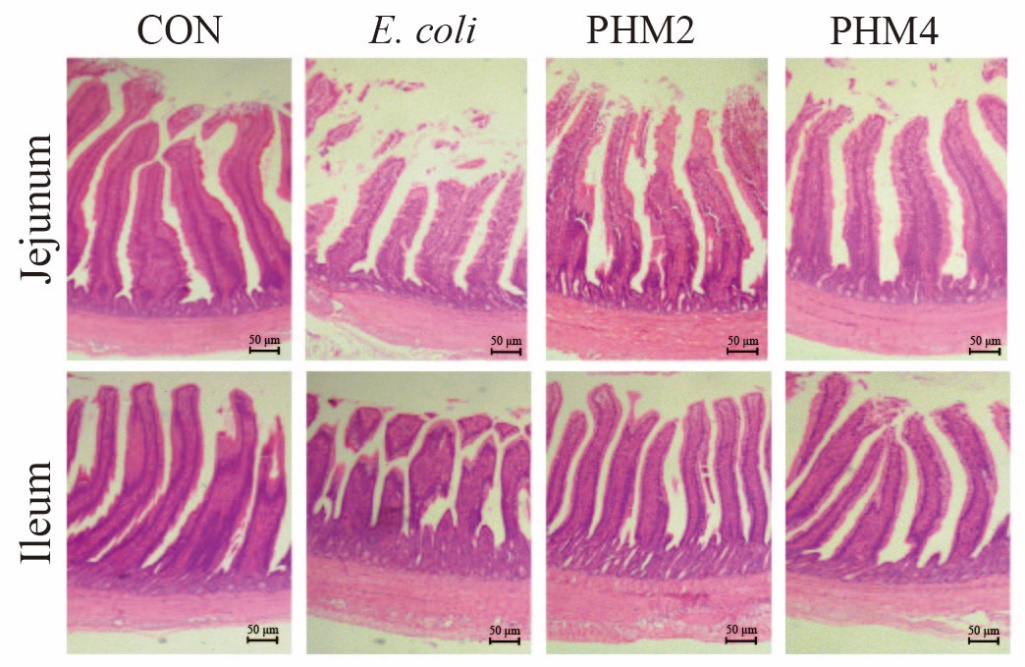


Figure S1. Effect of PHM on jejunal and ileal morphology in broilers infected with APEC by H&E staining (40×).
